# Use and Perceptions of Oncology CT Structured Reports in Australia and New Zealand

**DOI:** 10.1111/1754-9485.13860

**Published:** 2025-07-02

**Authors:** K. J. Brown, A. Agarwal, K. L. Gormly

**Affiliations:** ^1^ The Royal Adelaide Hospital South Australian Medical Imaging Adelaide South Australia Australia; ^2^ Jones Radiology South Australia Australia; ^3^ The University of Adelaide Adelaide Australia

**Keywords:** computational intelligence, diagnostic imaging, medical oncology, neoplasm, tomography

## Abstract

**Introduction:**

Structured oncology template (OT) reports are preferred by physicians. Promoted in Australia and New Zealand (ANZ) since 2008, this study investigates the current OT use in ANZ and perceptions around OT reporting.

**Methods:**

An online survey was created and sent to > 350 ANZ radiologists, aiming to gain insight into the current rates of OT reporting, demographic variations, OT types, implementation and perceived advantages and limitations. Statistical analyses were descriptive.

**Results:**

Of 164 respondents, 21% never used an OT; 49% used OTs for > 75% of oncology reports, highest rates were those with 10–20 years of experience (29/61) and reporting > 10 oncology CTs per week (10–20 cases 74% and > 20 cases 72%); 72% used tumour, lymph node and metastases headings; 57% used OTs for all diagnosed cancers; 37% used measurement tables; 70% used subjective terms in their conclusion; and only 4% used synoptic reports. Most selected advantage was ‘More clarity and increased communication with clinicians’. Most selected disadvantage was ‘lack of experience in template reporting’ for those using an OT and ‘Inflexible and limits creativity’ for those who did not.

**Conclusion:**

OT use is supported across ANZ. Use is higher in those reporting more oncology cases and with 10–20 years of experience. OT reports are perceived as beneficial by those using them, while those who do not perceive them as inflexible.

## 1 Introduction

In cancer imaging, the radiology report is a key form of communication between radiologists and clinicians, providing diagnostic and prognostic information used in making patient treatment decisions [1, 2, 3, 4]. Structured reports improve efficiency, clarity and completeness of reports and result in improved collaboration with other clinicians [2, 5]. They are proven to be predictable, reproducible, succinct and organised and are preferred by many clinicians and radiologists compared to traditional freestyle reports [6]. Freestyle reports do not always address key clinical questions, may contain errors and can use ambiguous language [7, 8]. In oncology, structured reports allow easy comparison between scans over time and at different institutions [9]. Over the past two decades, there has been a worldwide movement to encourage structured reporting among radiologists by national and international radiology colleges and speciality interest groups [4, 10, 11].

Structured, standardised or template reports are terms used to describe reports with a consistent order, which usually have subheadings and specified content [12]. The interchangeable use of these terms causes confusion. Specific definitions proposed by Nobel et al. restrict ‘standardisation’ to the report content, with consistent terms and use of a lexicon or uniform language, while structured reporting describes the layout and varying degrees of IT data entry [13, 14]. Level 1 structured reports are restricted to a consistent order of layout with subheadings, and Level 2 structured reports consist of predefined terminology and varying degrees of IT tick boxes and report generation [4, 13]. These definitions provide clarity to this area and this article follows the Nobel terminology for consistency [13].

In Oncology, Level 1 reports may be cancer specific, particularly for staging or following a generic tumour, lymph node and metastases order [15]. A Level 1 Oncology structured report template was published and promoted in Australia and New Zealand (ANZ) in 2008 for use with oncology CTs, particularly follow‐up studies. The Oncology Template (OT) with generic headings of tumours, lymph nodes and metastases with measurement tables was designed to improve consistency, clarity and communication between oncologists and radiologists [16]. The OT was supported and encouraged by oncologists [17]. An audit in 2009 comparing the use of the OT and free‐text reporting demonstrated a significant increase in the inclusion of measurements and conclusions when using the OT.

A 2021 survey of ANZ radiologists found that 72.5% of respondents work in a department using some form of structured reporting [12]. However, the rate of Level 1 oncology structured report use in ANZ is unknown. This study surveyed radiologists across ANZ to assess the use, format, perception and institutional factors of OT reports, 14 years after their introduction.

## 2 Methods

A survey was prospectively designed to obtain information regarding the current use of OT reports, structure, relation to voice recognition software availability, type of training provided, whether this was provided by the institutions or self‐initiated and current perceptions regarding advantages and disadvantages of OT use. This study was aimed at practising radiologists in ANZ. Demographic information, including country or territory of work, whether respondents worked in public, private or both, years of experience as a radiologist and frequency of oncology reporting, was also reported to assess for any association of those factors with the use of the OT. The questions and answers that could be selected are listed in the Supporting Information.

There were three locations along the survey path where respondents could be redirected to a different question. For example, if the answer to the question ‘How often do you use a template or structured report in your CT oncology reporting?’ was ‘Never’, the respondent was redirected away from questions that asked more detail about OT reporting to the question ‘Do you wish that your organisation implemented template reporting?’. Answer selection types were either ‘please select one’ or ‘please select all that apply’ with many questions having the option for further comment if the respondent wished to provide more details.

The survey was constructed on the SurveyMonkey platform and sent via an email link. A total of 342 radiologists were emailed via the Abdominal Radiology Group of Australia and New Zealand (ARGANZ) mailing list, with additional emails sent directly to consultant radiologists at main hospitals in each Australian state and NZ in June 2021. Radiologists who received the email were encouraged to forward the survey to colleagues, so the total number receiving the survey is unknown.

After the survey closure in May 2022, data were collated and analysed using descriptive statistics, Microsoft Excel (Microsoft Corporation, 2018) and online statistical calculators from Social Science Statistics.

Ethics approval was received from the St Andrews Hospital Human Research Ethics Committee. No patient data were collected and no patient consent was required.

## 3 Results

**FIGURE 1:**
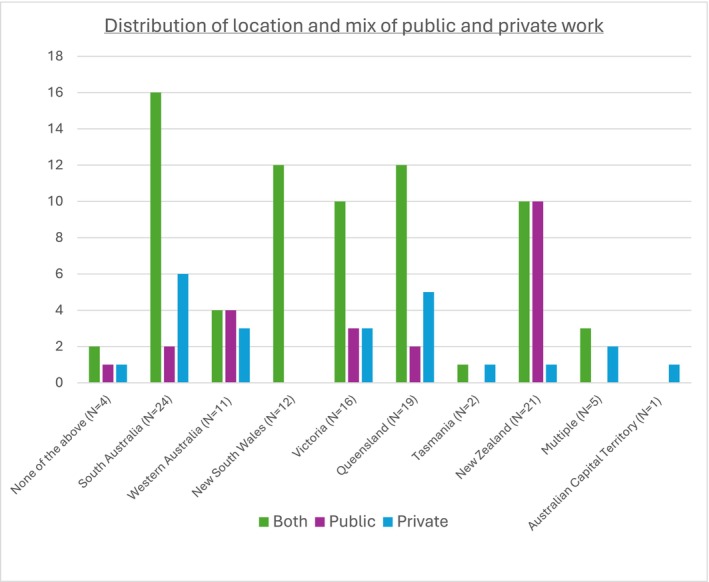
Distribution of location and mix of public and private work (*n* = 115).

A total of 165 radiologists answered the survey with an 88% completion rate. As not all radiologists answered each question, the described denominator indicates the number of radiologists who answered the question. One respondent's data was not included due to a survey error. The distribution of location and mix of public and private work is shown in Figure 1. There was a higher proportion of more experienced radiologists responding to the survey, with 107 having more than 10 years of experience and 57 having less than 10 years of experience (Table 1).

**TABLE 1:** Frequency of use of OT by number of years working as a radiologist, number of oncology scans reported per week and location of work.

Frequency of oncology template use	Never	< 25%	25%–75%	> 75%	Total
Year experience					
< 2 years	0	0	5	3	8
2–5 years	1	3	3	14	21
5–10 years	7	3	1	17	28
10–20 years	13	9	10	29	61
> 20 years	14	6	9	17	46
Unanswered	0	0	0	0	0
Oncology reports per week δ					
< 2 reports	N/A	8	0	3	11
2–5 reports	N/A	2	5	11	18
5–10 reports	N/A	1	7	15	23
10–20 reports	N/A	1	7	23	31
> 20 reports	N/A	3	6	23	32
Unanswered	N/A	6	3	5	14

*Note:* δ Only those who use template reports at least sometimes answered this question.

Abbreviation: OT, oncology template.

A total of 129/164 (79%) of the responding radiologists used templates at least occasionally in their daily work, with the frequency of OT use by years of experience and number of oncology reports per week displayed in Table 1. 49% (80/164) of radiologists used an OT > 75% of the time for oncological reports. 21% (35/164) never used templates. Of these, this was most common in the > 20‐year experience group at 30% (14/46).

Geographic variation in OT use is illustrated in Table 2. South Australia was the most likely location to use an OT, with 96% (24/25) reporting at least occasional OT use and Western Australia was the least likely location to use an OT, with a ‘never’ percentage of 41% (7/17).

**TABLE 2:** Distribution of the frequency of OT use by geographic location across ANZ.

State or territory	Never	< 25%	25%–75%	> 75%
None of the above	0	0	0	3
South Australia	1	0	0	24
Western Australia	7	4	3	2
Victoria	8	5	6	7
New South Wales	8	2	4	10
Queensland	5	4	4	12
Tasmania	1	1	2	0
Australian Capital Territory	0	1	0	0
New Zealand	4	3	6	17

Abbreviation: OT, oncology template.

The frequency of use of OT for different types of cancer patients is shown in Table 3. Five of 115 (4%) used synoptic reports with drop‐down boxes (Level 2 structured report) for CT oncology reporting.

**TABLE 3:** Frequency of use of OT versus type of patient OT is used for.

	Trial patients	All diagnosed cancers	Specific cancer	Other
< 25%	6	5	6	1
25%–75%	8	19	4	0
> 75%	23	69	5	4

Abbreviation: OT, oncology template.

A total of 83/115 (72%) used ‘Primary Tumour, Lymph nodes, Metastases and other findings’ as subheadings and 31/115 (27%) had other subtitle variations. Four per cent (5/115) commented that they preferred to use anatomical or body regions, and one radiologist commented that it depends on whether the patient was in a trial or not.

Measurement tables were used by 43/115 (37%). Tables were more frequently used by radiologists who use OT > 75% of the time and those who report > 20 oncology studies per week at 72% (31/43) and 53% (17/32), respectively.

Eighty‐three per cent (95/115) of respondents regularly measure ≤ 5 lesions, 17% (19/115) measure 6–8 lesions and 1 respondent routinely measures > 8 lesions. New Zealand radiologists had a higher proportion of > 5 lesion measurements compared to Australian states and territories, at 33% (7/21).

The terminology used in the conclusion is shown in Table 4, with one respondent not providing a conclusion.

**TABLE 4:** The types of terminology used in oncology report conclusions, noting respondents were able to select more than one answer.

Subjective assessment	80/114
RECIST terms	48/114
% Change since previous	12/114
No conclusion	1/114

Seventy‐six per cent (87/115) had voice recognition (VR) available at their institution, and 62% (54/87) had templates integrated into the VR software, the highest percentage having it implemented for 2–5 years (22/50, 44%). For those without a template integrated, the majority had templates readily available. Of those who never used templates, and those who did but do not have them readily available, 42% (16/38) wished their organisation implemented templates. 90% (45/50) of respondents stated they learnt how to use templates by self‐initiated learning or learning on the job.

**FIGURE 2:**
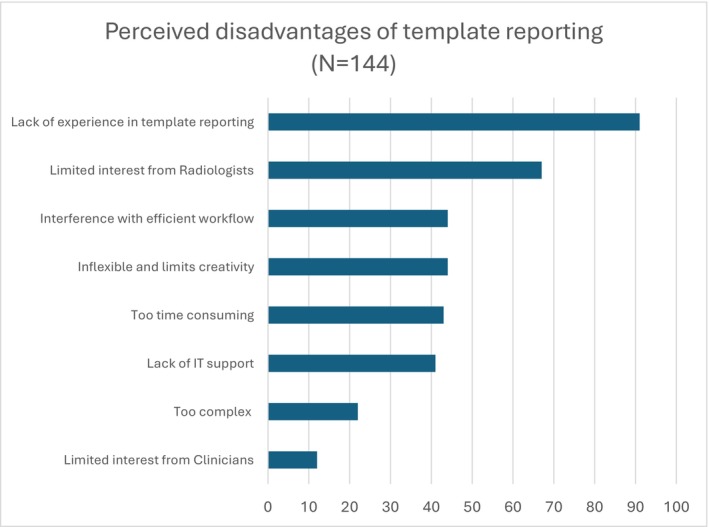
Perceived advantages of template reporting.

**FIGURE 3:**
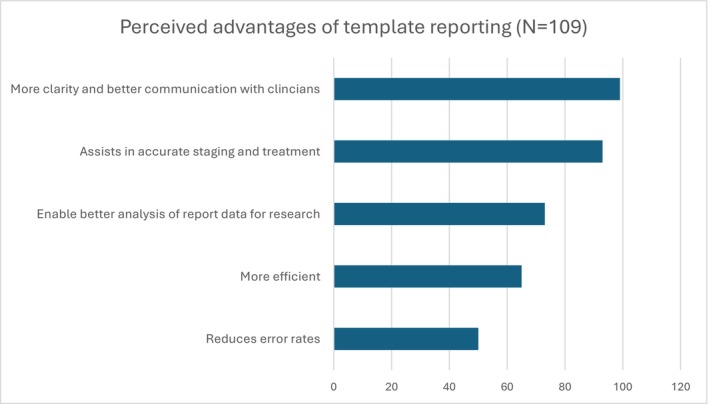
Perceived disadvantages of template reporting.

Perceived advantages and disadvantages are shown in Figures 2 and 3. Additional comments regarding the perceived advantages of OT included ‘Makes comparing past reports much easier’ and ‘Allows more consistent follow up’. The most common disadvantage for those using an OT was ‘lack of experience in template reporting’, but those not using an OT selected ‘Inflexible and limits creativity’. Comments on perceived disadvantages to OT included ‘many radiologists are resistant to changing ingrained practice and unwilling to take time to learn new skills’, ‘training’, ‘old and lazy’ and ‘some RIS don't allow tables’. Amongst radiologists who never use an OT, comments included ‘My reporting is always in a certain order… The template reports disrupt that so I sometimes feel like I haven't looked at a scan properly’ and ‘if a referrer were to give me an oncology template, I would use it. I presume there is a good reason that they never do’. Some indicated a wish to change with comments such as ‘I used to not like template reporting but with the volume of oncology imaging growing so much I am definitely seeing the usefulness and consider changing my practice’.

## 4 Discussion

Around the world, there has been growing support for structured reporting, particularly by large international societies, such as the European Society of Radiology and the Radiological Society of North America [6, 10, 18]. Their use has been made easier by the success of standardised terminology with lexicons such as BIRADS, Lung‐RADS, ORADS and TIRADs [14, 19, 20, 21, 22]. However, there is variable uptake and moderate levels of resistance to commencing structured reporting by individual radiologists [23]. In Oncology imaging, structured reports provide ease of comparison to baseline and/or previous imaging studies [20, 24]. The basic Level 1 OT assessed in this paper was specifically designed in 2008 for flexibility across multiple cancers for staging, particularly follow‐up studies. It encouraged radiologists to report in a clinical order by first addressing the primary tumour and spread, with inclusion of definitive statements regarding the presence or absence of lymph nodes and metastases, inclusion of measurements and a conclusion.

In ANZ, radiologists report oncology scans in various settings, including cancer centres, public hospitals and private institutions, and have a wide range of experience. The spread of numbers of weekly oncology reports by radiologists in this survey supports the significant variation in experience of radiologists reporting these studies in both the public and private sectors. Higher volume oncology CT reporters were more likely to use an OT and tables. This could be for a number of reasons, but it supports the idea that frequent users appreciate benefits such as improving communication with both clinicians and future reporting radiologists, easier reporting of follow‐up studies when the past report is easy to read, containing relevant measurements, and a time advantage when used regularly. In addition to reproducibility and easy readability, template reports collate key information often sought by researchers at a later date. Although the work of the reporting radiologist may, perhaps unfairly, go unacknowledged in resultant manuscripts, the information included in the template report provides the added benefit of reducing the workload of research teams. Across all radiologists answering the survey, most thought that structured reports result in better clarity, improved communication with clinicians and assist in accurate staging and treatment, which is consistent with the current literature [5, 24, 25]. It could be argued that those reporting oncology scans less frequently may benefit even more from the prompts associated with a structured report.

Interestingly, there were differences in perceived barriers between radiologists who do and do not use structured reports. Amongst those who do use OT reports, the most frequent barrier was ‘lack of experience in template reporting’, whereas radiologists who do not use an OT selected ‘Inflexible and limits creativity’ more frequently. The latter reason is similar to the survey performed by Faggioni et al., where ‘excessive report simplification’ and ‘template rigidity’ were the top two perceived weaknesses [26]. Radiologists carefully consider what enters their reports, so that it is individualised and personal [6]. Furthermore, the act of dictating in a narrative form may be fundamental to the cognitive processing of the case [27]. However, given the movements towards increasing standardisation and AI assistance, such a level of individual autonomy may become undesirable for optimal patient care. Level 1 structured reports retain a certain amount of free text, allowing the radiologist the ability to express their assessment, but also provide the clinician and following radiologist a consistent structure for ease of reading. Some radiologists in our survey who never use templates believed that templates are ‘Too time consuming’. However, while some studies claim that report template usage is more time consuming, a systematic review by Nobel et al. shows that most studies find a reduction in dictation time [28].

Our survey demonstrated locoregional variation in the use of OT, with South Australian radiologists being the most likely group to use an OT. This is likely due to extra training in South Australia, which is the location of the radiologist involved in designing and promoting OT use. Single education sessions were run in NSW, QLD, WA and NZ in 2009–2012, but following this, training relied on a local champion to promote and encourage OT use. In our survey, most radiologists (47/50) learnt how to use OT by self‐initiated learning or learning on the job, such as from other people's reports, suggesting that there is currently limited training during registrar years and in departments. However, survey respondents who use OT > 75% of the time were more likely to be in the 2–5 year experience group, which may be due to exposure during training years, which may indicate increasing OT acceptance. In 2022, Pool et al. found that 39% (35/89) thought that education and experience using structured reports would help make radiologists use structured reporting more in their practice [12]. Indeed, limited formal training in template reporting has been documented as a reason for reduced use; however, aversion to structural reporting tends to fade with subsequent introduction of formal teaching [24].

The majority of radiologists using a Level 1 OT followed the structure of primary tumour, lymph nodes and metastases described in the original paper [16]. Additional separate headings included nontarget lesions and target lesions. Measurement tables were less frequently used, although all respondents to that question indicated they measured lesions. This may be due to tables not being considered a useful or efficient way to present findings and/or IT challenges, as tables are not supported by many RIS systems.

For the report conclusion, 70% reported using subjective descriptors and 42% RECIST terms, with a smaller proportion providing a percentage change in summated diameters. While using RECIST terms outside of a trial setting is not specifically recommended, there is a risk of ambiguity and misinterpretation associated with subjective terms. A percentage change since previous is the most objective measure, noting it does not capture changes in morphology, but was the least frequent method reported. The variability in responses across both trial and nontrial patients and respondents selecting multiple answers supports the fact that there is no universally agreed conclusion method for general oncology follow‐up reporting.

Despite a number of perceived barriers, 31% of those who never use OT reports indicated a wish for their organisation to implement them. The concern of an OT causing ‘Interference with efficient workflow’ suggests that template integration into the RIS/PACS system is integral to the success of OT's widespread use.

Our results have identified multiple areas that can be addressed to improve the uptake of structured reporting in radiology. At departmental level, a combination of proactive training of registrars and radiologists, and encouragement or expectation of use within departments, may assist more radiologists in starting to use templates and, subsequently, overcoming perceived obstacles. Uploading templates into dictation systems by departments reduces individual barriers to setup. Having dictatable fields increases efficiency and prevents distraction of the radiologist by having to click on fields or select boxes. Full integration of templates into systems can allow auto‐population of the study type, clinical information, type and dose of contrast and technique, which saves time. Further advances include measurements automatically integrated into the report [18]. Calculation of tumour response using RECIST or other commonly used criteria could be considered. A detailed guide to clinical implementation is documented by Jorg et al., however, it requires a substantial effort for organisations to implement [24].

More centralised incentives could also be considered. In Australia in 2022, the National Pathology Accreditation Advisory Council of the Royal College of Pathologists of Australia mandated that all cancer pathology reports for which they had produced a structured report must be reported using that template [29]. Some countries have devised national initiatives in radiology, such as Germany, which has an IT subcommittee actively developing national templates that are available on a dedicated website. Similar initiatives are present in Italy, Switzerland and North America [18]. Other countries show support at a national level, but implementation is up to the individual or the department. Centralised roll‐out with VR platforms would be beneficial.

## 5 Limitations

Our study is limited by the moderate number of respondents who were recruited primarily from a subspecialty abdominal imaging society and thus are not representative of the general population of radiologists. The nature of selecting a survey to assess radiologists carries the risk of a ‘non‐response’ bias.

On the SurveyMonkey platform, one radiologist was allowed to bypass the redirects ingrained in the survey, which is a suspected IT error.

Although we assessed the years of practising as a consultant, we did not assess the years of exposure or use of OT, which would have been a useful addition to the survey. Also, it would have been interesting to know how many reports are produced by the respondents who never use OT; however, in the study design, these radiologists were redirected away from this question.

## 6 Conclusion

Level 1 structured reports for oncology imaging are currently being used throughout Australia and New Zealand, with low rates of Level 2 structured reports. Use is more frequent in those reporting more oncology cases. Radiologists appreciate the advantages of OT, including referrer preference, improved clarity, improved communication and better accuracy, with the perception of inflexibility more common in those not using them. Lack of experience and training were identified as areas that could be addressed to increase use.

## Ethics Statement

This study was approved by the St Andrew's Hospital Human Research Ethics Committee.

## Conflicts of Interest

The authors declare no conflicts of interest.

## Supporting information


Appendix S1.

## Data Availability

The data that support the findings of this study are available from Jones Radiology. Restrictions apply to the availability of these data, which were used under license for this study. Data are available from the author(s) with the permission of Jones Radiology.
