# Supplementary material for: Use and Perceptions of Oncology CT Structured Reports in Australia and New Zealand
Source: J Med Imaging Radiat Oncol. 2025 Jul 2;69(5):554–60. doi: 10.1111/1754-9485.13860 (PMC12317362; doi:10.1111/1754-9485.13860)
Supplement: Supplementary file 1 — Appendix S1. [file ARA-69-554-s001.pdf]

SURVEY QUESTIONS

	Question	Type of answer selection	Answers
1	Where do you work?	Please select all that apply	None of the above
			South Australia
			Western Australia
			New South Wales
			Victoria
			Queensland
			Tasmania
			Australian Capital Territory
			Northern Territory
			New Zealand
			Multiple
			Other (please specify)
2	How many years of experience do you have as a Radiologist?	Select one answer	< 2 years
			2-5 years
			5-10 years
			10-20 years
			More than 20 years
3	How often do you use a template or structured report in your CT oncology reporting? *	Select one answer	Never
			< 25%
			25-75%
			> 75%
4	Do you use CT template oncology reporting in...	Select one answer	Private
			Public
			Both
5	How many Oncology template reports would you perform per week?	Select one answer	< 2
			2-5
			5-10
			10-20
			> 20
6	Are the templates used for...?	Please select all that apply	Trial patients
			All diagnosed cancers
			Only for specific cancers
			Other (please specify)
7	What is most similar to your subheadings in CT template reports?	Select one answer	Primary, Lymph nodes, Metastases and Other Findings.
			Primary, Target lesions, Non-target lesions and Other Findings
			Primary, Lymph nodes, Metastases, Non-target lesions and Other Findings.
			None of the above
			Other (please specify)
8		Select one answer	Yes

	Are measurement tables included in your CT oncology template reports?		No
			Other (please specify)
9	How many lesions do you typically measure?	Select one answer	< or = 5
			6-8
			>8
			None of the above
10	Do the CT oncology templates allow free text options?	Select one answer	Yes
			No
			Other (please specify)
11	How do you usually indicate disease response in your conclusion?	Please select all that apply	RECIST terms
			% change since previous scan
			subjective assessment (e.g. no change, improvement or progression/worsening)
			I don't specify
			I don't provide a conclusion
12	Do you know what RECIST 1.1 reporting criteria are?	Select one answer	Yes
			No
13	Do you use a cancer specific CT oncology reporting template or structured report?	Select one answer	Yes. Please state which.
			No
14	Do you use synoptic CT oncology reports (e.g. drop down boxes with no free text) with pre-selected options?	Select one answer	Yes
			No
15	Do you use template or synoptic reporting for other modalities?	Please select all that apply	MRI
			PET
			US
			None
16	Does your organisation use voice recognition software?**	Select one answer	Yes
			No
17	Are templates integrated into the Voice Recognition software?***	Select one answer	Yes
			No
18	How long has your organisation had TNM Template reporting integrated into the PACS reporting system?	Select one answer	< 2 years
			2-5 years
			6-10 years
19		Select one answer	Yes

	Did you undergo any training for use of the template system?		No
			Other (please specify)
20	How did you learn to use the template Oncology reporting?	Please select all that apply	Self initiated learning about TNM/RECIST
			Learning on the job ie. from other peoples reports
			Still unsure of how to use the template
			My institution provided a training session
			My institution provided training materials
			Other:
21	In your opinion, what are the barriers to Radiologists issuing TNM template based reports?	Please select all that apply	Too time consuming
			Lack of experience in template reporting
			Lack of IT support
			Inflexible and limits creativity
			Limited interest from Radiologists
			Limited interest from Clinicians
			Too complex
			Interference with efficient workflow
			Additional comments
22	What do you consider are the benefits of oncology template reporting? (tick all that apply)	Please select all that apply	Assists in accurate staging and treatment
			More efficient
			More clarity and better communication with clinicians
			Enable better analysis of report data for research
			Reduces error rates
			Additional comments

*if the answer was ‘no’ respondents were re-directed down pathway 1
** if the answer was ‘no’ respondents were re-directed down pathway 2
*** if the answer was ‘no’ respondents were re-directed down pathway 3

Pathway redirect 1

4	Do you wish that your organisation implemented template reporting?	Please select one option and optional comment	Yes
			No
			Comment
5			Too time consuming

	In your opinion, what are the barriers to Radiologists issuing TNM template based reports?	Please select all that apply	Lack of experience in template reporting
			Lack of IT support
			Inflexible and limits creativity
			Limited interest from Radiologists
			Limited interest from Clinicians
			Too complex
			Interference with efficient workflow
			Additional comments
6	What do you consider are the benefits of oncology template reporting? (tick all that apply)	Please select all that apply	Assists in accurate staging and treatment
			More efficient
			More clarity and better communication with clinicians
			Enable better analysis of report data for research
			Reduces error rates
			Additional comments

Pathway redirect 2

17	How did you learn to use the template Oncology reporting?	Please select all that apply	Self initiated learning about TNM/RECIST
			Learning on the job ie. from other peoples reports
			Still unsure of how to use the template
			My institution provided a training session
			My institution provided training materials
			Other:
18	In your opinion, what are the barriers to Radiologists issuing TNM template based reports?	Please select all that apply	Too time consuming
			Lack of experience in template reporting
			Lack of IT support
			Inflexible and limits creativity
			Limited interest from Radiologists
			Limited interest from Clinicians
			Too complex
			Interference with efficient workflow
			Additional comments

19	What do you consider are the benefits of oncology template reporting? (tick all that apply)	Please select all that apply	Assists in accurate staging and treatment
			More efficient
			More clarity and better communication with clinicians
			Enable better analysis of report data for research
			Reduces error rates
			Additional comments

Pathway Redirect 3

18	If no, are the template reports readily accessible?	Please select one	Yes
			No
19	Did you undergo any training for use of the template system?	Select one answer	Yes
			No
			Other (please specify)
20	How did you learn to use the template Oncology reporting?	Please select all that apply	Self initiated learning about TNM/RECIST
			Learning on the job ie. from other peoples reports
			Still unsure of how to use the template
			My institution provided a training session
			My institution provided training materials
			Other:
21	In your opinion, what are the barriers to Radiologists issuing TNM template based reports?	Please select all that apply	Too time consuming
			Lack of experience in template reporting
			Lack of IT support
			Inflexible and limits creativity
			Limited interest from Radiologists
			Limited interest from Clinicians
			Too complex
			Interference with efficient workflow
			Additional comments
22	What do you consider are the benefits of oncology template reporting? (tick all that apply)	Please select all that apply	Assists in accurate staging and treatment
			More efficient
			More clarity and better communication with clinicians

			Enable better analysis of report data for research
			Reduces error rates
			Additional comments
